# Contagion Dynamics for Manifold Learning

**DOI:** 10.3389/fdata.2022.668356

**Published:** 2022-04-26

**Authors:** Barbara I. Mahler

**Affiliations:** Mathematical Institute, University of Oxford, Oxford, United Kingdom

**Keywords:** dimensionality reduction, manifold learning, topological data analysis, persistent homology, contagion

## Abstract

Contagion maps exploit activation times in threshold contagions to assign vectors in high-dimensional Euclidean space to the nodes of a network. A point cloud that is the image of a contagion map reflects both the structure underlying the network and the spreading behavior of the contagion on it. Intuitively, such a point cloud exhibits features of the network's underlying structure if the contagion spreads along that structure, an observation which suggests contagion maps as a viable manifold-learning technique. We test contagion maps and variants thereof as a manifold-learning tool on a number of different synthetic and real-world data sets, and we compare their performance to that of Isomap, one of the most well-known manifold-learning algorithms. We find that, under certain conditions, contagion maps are able to reliably detect underlying manifold structure in noisy data, while Isomap fails due to noise-induced error. This consolidates contagion maps as a technique for manifold learning. We also demonstrate that processing distance estimates between data points before performing methods to determine geometry, topology and dimensionality of a data set leads to clearer results for both Isomap and contagion maps.

## 1. Introduction

Manifold-learning techniques aim to identify low-dimensional manifold structure in high-dimensional data (Lee and Verleysen, 2007). High-dimensional point-cloud data may represent a large number of features on a collection of objects. Some of these features may be redundant or irrelevant, thus giving the data lower-dimensional intrinsic structure. Alternatively, high-dimensional point-cloud data with low-dimensional intrinsic structure may arise as a sample of points from a low-dimensional manifold that is embedded in a high-dimensional space.

Consider, for instance, data points that lie on a plane in three-dimensional space. Principal component analysis (PCA), a classical dimensionality-reduction technique (Sorzano et al., 2014), can find the directions along which the data has maximum variance as well as the relative importance of these directions. In the case of the plane embedded in three-dimensional space, PCA returns three vectors: two of positive weight spanning the plane and one vector of zero weight that is orthogonal to the plane. PCA can thus identify the plane underlying the ostensibly three-dimensional data. More generally, consider data points that are concentrated around a low-dimensional manifold (reflecting the underlying information) that is embedded in a high-dimensional space. PCA is a *linear* dimensionality-reduction method: If the manifold is non-linear, PCA is unable to detect the low-dimensional space underlying the data set. This is where manifold-learning techniques (as a type of *non-linear* dimensionality reduction) can be effective. The purpose of manifold learning is to uncover low-dimensional manifold structure of a data set in a high-dimensional feature space, even if the structure of the data is curved.

A common procedure for non-linear dimensionality reduction is the following:

1. Create a network on the data points, such as by defining an edge between any two nodes within some distance ϵ (producing the ϵ*-neighborhood graph*) or by connecting each point to its *k* closest neighbors (producing the *k-nearest-neighbor graph*).
2. Define some notion of distance between data points based on this network [e.g., shortest-path length for Isomap (Tenenbaum et al., 2000)]. The aim is to approximate the actual geodesic distance on the underlying manifold.
3. Map points to some space based on the pairwise distances. Possible ways of doing this include the following: (a) use a multidimensional scaling algorithm, which finds an embedding that preserves pairwise distances as well as possible; or (b) take distances to be coordinates in a space of dimension equal to the number of data points [the approach that contagion maps take as a manifold-learning technique (Taylor et al., 2015)].

Many well-established manifold-learning techniques perform poorly when faced with noisy data. Isomap, for instance, can be very sensitive to noise. Consider, for example, a noisy point sample on the Swiss roll (see e.g., **Figure 2A**). Noise can lead to two points on adjacent sheets lying close together. The *k*-nearest-neighbor graph might then have an edge that connects the corresponding nodes (i.e., a “short-circuit error”), although the points lie far apart in the intrinsic geometry. Consequently, Isomap falsely considers the two points to be close, and it thus fails as a manifold-learning technique in this case.

Contagion maps (Taylor et al., 2015) can circumvent Isomap's “short-circuit error” issue by exploiting the “social reinforcement” phenomenon that characterizes threshold contagions. When the threshold of a contagion is small enough to allow spreading *via* a single edge, the associated contagion map can be viewed as a variant of Isomap and is similarly sensitive to the type of noise described above. For larger thresholds, however, a single errant edge in the *k*-nearest-neighbor graph cannot carry a contagion, and, as a result, the contagion map does not view the two points as close and performs well as a manifold-learning technique. When a contagion on a network spreads as a wavefront exclusively *via* edges between nodes that are close together in the intrinsic geometry of the underlying domain, we call this *wavefront propagation* (WFP). When it spreads *via* edges that connect nodes that are far apart from each other in the underlying domain, thereby creating new contagion clusters in regions of the network that are far from the previously infected regions, we call this *appearance of new clusters* (ANC). If a contagion spreads predominantly *via* WFP, then, intuitively, the point cloud that is the image of the corresponding contagion map exhibits features of the network's underlying structure, and this has been confirmed for particular classes of networks in Taylor et al. (2015) and Mahler (2021). This recovery of underlying structure under certain spreading dynamics suggests contagion maps as a manifold-learning technique.

We use persistent homology, a method from topological data analysis (Edelsbrunner and Harer, 2008), as well as more established statistical techniques to perform manifold learning based on contagion dynamics, and we compare this approach to Isomap-type algorithms. One of the most common applications of persistent homology is the task of recovering a manifold from a random, potentially noisy sample of points (Carlsson, 2009). This application illuminates the natural overlap of topological data analysis with manifold learning. Both are designed to find shape regardless of exact geometry (including measures of curvature and length), and both aim to be robust to noise. Traditionally, they differ in their respective approaches and, as a result, in their ability to identify different types of structural features. Persistent homology can, for example, identify a sphere (by its topological features in dimensions 1 and 2), but not a Swiss roll (as it has no non-trivial topological features). A manifold-learning algorithm like Isomap, on the other hand, can “unroll” the Swiss roll (under favorable conditions), but cannot see that a sphere is a two-dimensional manifold: Isomap detects a sphere's lowest embedding dimension 3, but cannot see its intrinsic 2-dimensional structure. In this article, we combine the two approaches by processing our data *via* a manifold-learning-type procedure first and then computing persistent homology (along with some other measures) based on this processed data.

The manifold-learning-type procedure that we use is based either on activation times in a threshold contagion or on shortest-path distances between nodes in a network built on the given data. In both cases, we compare two different approaches: We either pass the distance estimates directly into a pipeline for analyzing dimensionality, topology, and geometry, or we first process them to create points in high-dimensional space, whose pairwise distances we then pass into the same pipeline. We not only compare the contagion-based approach to the Isomap-type one, but also examine the effect of this pre-processing step on our results. We find that the contagion-based approach proves successful in many cases where the Isomap-type approach breaks down and that the pre-processing step leads to clearer results in general.

## 2. Materials and Methods

The fundamental hypothesis that our algorithms are built on is that our data come as samples from some underlying submanifold of ℝ^*n*^, which we want to infer. To this end, we perform variants of a procedure whose basic steps are as follows.

First, if a data set is given in the form of a point cloud, we start by constructing a *neighborhood graph* (*V, E*) based on this point cloud. We do so by associating a set *V* of nodes to the data points (denoting by *i* the node associated to point *p*_*i*_) and defining the edge set *E* to build either (1) a *k-nearest-neighbor graph* by connecting each point to its *k* closest neighbors, or (2) an ϵ*-neighborhood graph* by connecting any two points that are within ϵ from one another:

1. Given some *k* ∈ ℕ, we have (*i, j*) ∈ *E* if and only if *p*_*i*_ is in the set of the *k* nearest neighbors of *p*_*j*_ or vice versa.
2. Given some ϵ ∈ ℝ_>0_, we have (*i, j*) ∈ *E* if and only if *d*_2_(*p*_*i*_, *p*_*j*_) ≤ ϵ.

In both types of neighborhood graph, one can either weight the edges by the corresponding pairwise distances or treat the edges as unweighted^1^.

If we are given data in the form of a network, we use this given (weighted or unweighted) network instead.

Second, we calculate a notion of distance between points that is aimed to be an estimate for the actual geodesic distance on the underlying manifold. The idea is that only the pairwise Euclidean distances between neighboring pairs of points approximate the geodesic distances sufficiently, and that estimates for geodesic distances between non-neighboring pairs of points can be inferred from the distances between neighboring points by “tracing” through the neighborhood graph.

The precise pairwise distances between adjacent points in a neighborhood graph provide information that is relevant to estimating the geodesic distances between non-neighboring points. Unweighted neighborhood graphs forget this information and thus tend to lead to less accurate approximations to the geodesic distances. The loss of information from taking an unweighted graph can be greater for *k*-nearest-neighbor graphs than for ϵ-neighborhood graphs, because, in the latter case, the pairwise distances are within a range that is capped by ϵ.

We examine dimensionality, topology, and geometry as follows. We perform classical multidimensional scaling (MDS) (Torgerson, 1958) based either on the dissimilarity measure (i.e., activation times in the case of contagion-based algorithms, and shortest-path lengths in the case of Isomap-type algorithms) directly or on the Euclidean distances between points in high-dimensional space whose coordinates are the distance estimates. MDS aims to embed a point cloud in a given low-dimensional vector space in a way that preserves given distances between pairs of points as well as possible. It does so by minimizing a cost function called “strain”: Given a matrix *D* = (*d*_*ij*_)_*i,j*∈*I*_ of pairwise distances, or “dissimilarities” (not necessarily satisfying the defining properties of a metric), and some Euclidean target space *Y*, MDS finds coordinate vectors {*y*_*i*_ ∈ *Y*}_*i*∈*I*_ that minimize the cost function


$$E=\|\tau \left(D\right)-\tau \left(D_{Y}\right)|{|}_{L^{2}},$$

where *D*_*Y*_ = (‖*y*_*i*_ − *y*_*j*_‖_2_)_*i,j*∈*I*_, ${\left\|M\right\|}_{L^{2}}=\sqrt{\sum_{i,j}M_{ij}^{2}}$, and τ(*M*) = −*HSH*/2 with $S_{ij}=M_{ij}^{2}$ and *H*_*ij*_ = δ_*ij*_ − 1/|*I*| (Mardia et al., 1979).

MDS is a linear dimensionality-reduction technique when applied to Euclidean distances. By applying it to sensible approximations to the geodesic distances between data points, we hope to recover potentially curved structure. In other words, we hope to use MDS to achieve non-linear dimensionality reduction.

Given an embedding *via* MDS to Euclidean space of dimension *p*, we calculate its *residual variance* (Cox and Cox, 2010),


$$\begin{array}{l} R_{p}=1-{\left(\rho^{\left(p\right)}\right)}^{2}, \end{array}$$

where ρ^(*p*)^ is the Pearson correlation coefficient (Pearson, 1895) between the given pairwise distances {*d*_*ij*_}_*i,j*∈*I*_ and the corresponding pairwise Euclidean distances {‖*y*_*i*_−*y*_*j*_‖_2_}_*i,j*∈*I*_ between points in the embedding.

We determine the *approximate embedding dimension* *P* of the data (according to the given pairwise distances) by finding the smallest dimension such that the residual variance of the embedding *via* MDS to that dimension is less than 5%, that is,


$$\begin{array}{l} P=\min{\left\{p\;|\;R_{p}<0.05\right\}.}^{2} \end{array}$$

In addition to these dimensionality considerations *via* MDS, we analyze our data topologically by computing the persistent homology of the Vietoris–Rips filtration (Ghrist, 2008) based either on the dissimilarity measure directly or on the Euclidean distances between points in the associated high-dimensional point cloud. When a base-geometry is given, we also examine our data geometrically through a Pearson correlation coefficient between that base-geometry and the given dissimilarity measure. Note that such a known base-geometry to compare our processed data to is not usually given in manifold-learning applications. The measure is, however, useful when testing the algorithm on benchmark data.

When analyze the point cloud given by the columns of *D*, we are essentially applying our methods to a dissimilarity matrix that encodes the pairwise distances between the column vectors of *D*. We denote the operator that maps *D* to that matrix as


$$p_{\text{dist}}\;:\;M_{m,n}\left(\mathbb{R}\right)\to M_{n,n}\left(\mathbb{R}\right),$$

with (*p*_dist_(*D*))_*ij*_ = *d*_2_(D_*i_, D_*j_).

The methods for analyzing dimensionality, topology, and geometry described above were first used in tandem in Taylor et al. (2015) on contagion maps. That is, they were used after applying *p*_dist_ to a matrix holding the activation times in multiple realizations of a threshold contagion.

### 2.1. Contagion Maps

First, given point-cloud data, we obtain a neighborhood graph as described above. If the given data is a network, we work with this network directly instead. We denote the graph by (*V, E*), the number of nodes (i.e., the number of points in the case of point-cloud data) by |*V*| = *N*, and the graph's binary adjacency matrix by *A*. In order to get an estimate for the intrinsic distance between pairs of points, we then consider a threshold contagion on this network. We denote the state of node *i* ∈ *V* at time *t* by η_*i*_(*t*), which takes the value 1 if it is *active* and the value 0 if it is *inactive*. Given a set of seed nodes consisting of a node *j* ∈ *V* together with its immediate neighbors


$$S^{\left(j\right)}=\left\{j\right\}\cup \left\{k\;|\;A_{jk}\neq 0\right\}$$

that are active at time *t* = 0, and a threshold *T*, we update node states synchronously in discrete time steps according to the following rule. If η_*i*_(*t*) = 1, then η_*i*_(*t* + 1) = 1. If η_*i*_(*t*) = 0, then


$$\begin{array}{r} \eta_{i}\left(t+1\right)=1\;\text{if and only if }f_{i}>T,\\ \text{where }f_{i}=\frac{1}{d}\underset{k\in V}{\sum }A_{ik}\eta_{k}\left(t\right),\text{and }d\text{is the node degree.} \end{array}$$

Given a network (*V, E*) and a threshold *T*, a contagion seed yields a deterministic process, which we call a *realization* of the contagion model with *T* on (*V, E*). The activation time of node *i* in the realization seeded around node *j* is the smallest *t* such that η_*i*_(*t*) = 1, and we denote it by $x_{j}^{\left(i\right)}$. If node *i* is never activated in the realization that is seeded around node *j* ∈ *V*, we set $x_{j}^{\left(i\right)}=2N$ (i.e., larger than any actual activation time).

One can now work directly with this set of activation times, that is, treat the activation times as estimates for the geodesic distance between points on the underlying manifold, and use them to examine geometry, topology, and dimensionality of the data. Alternatively, one can first apply *p*_dist_. That is, one can work with points whose coordinate vectors are given by the columns of the dissimilarity matrix $D_{\text{cont}}={\left(x_{i}^{\left(j\right)}\right)}_{i,j\in V}$ that holds the activation times (or of a symmetrization of this dissimilarity matrix given by $D_{\text{cont}}+{\left(D_{\text{cont}}\right)}^{\text{T}}$). Using terminology from Taylor et al. (2015), producing such a point cloud is equivalent to mapping the nodes *via* a *contagion map*. The *regular contagion map* associated to (*V, E*) and *T* is the function from the set *V* of nodes to ℝ^*N*^ that is defined by


$$\begin{array}{l} i\mapsto x^{\left(i\right)}={\left[x_{1}^{\left(i\right)},x_{2}^{\left(i\right)},\ldots ,x_{N}^{\left(i\right)}\right]}^{T}. \end{array}$$

Similarly, the *symmetric contagion map* associated to (*V, E*) and *T* is the function from the set *V* of nodes to ℝ^*N*^ that is defined by


$$\begin{array}{l} i\mapsto {\left[x_{1}^{\left(i\right)}+x_{i}^{\left(1\right)},\ldots ,x_{N}^{\left(i\right)}+x_{i}^{\left(N\right)}\right]}^{T}. \end{array}$$

In this article, we work with symmetric contagion maps exclusively.

We examine dimensionality, topology, and geometry based on the activation times directly, that is, based on the entries of the matrix $D_{\text{cont}}+{\left(D_{\text{cont}}\right)}^{\text{T}}$, as well as after applying *p*_dist_ (i.e., we analyze the symmetric contagion map).

### 2.2. Isomap

The Isomap algorithm (Tenenbaum et al., 2000) is essentially a combination of a shortest-path algorithm with MDS. In a sense, Isomap works as a “non-linear version” of MDS which accommodates for potential curvature of data by incorporating a shortest-paths algorithm to estimate geodesic distances between data points.

The original Isomap algorithm proceeds as follows. First, given point-cloud data, Isomap starts by building a neighborhood graph (*V, E*) on the point cloud, as described at the beginning of section 2. Next, Isomap calculates the shortest-path lengths between pairs of nodes in this network using some shortest-path algorithm. We use the Floyd–Warshall algorithm (Floyd, 1962; Warshall, 1962) in this work. The set of shortest-path lengths can be recorded in a *dissimilarity matrix* *D*_iso_ = (*d*_G_(*i, j*))_*i,j*∈*V*_^3^, to which Isomap finally applies MDS to map the data points to a low-dimensional space.

As in the case of contagion maps, if data is given in the form of a network, we will work with this given network instead of a neighborhood graph. Moreover, in addition to the original Isomap algorithm, which simply projects points *via* MDS based on the set of shortest-path lengths (i.e., the entries in *D*_iso_), we calculate the residual variances of these projections, and we also examine this set topologically (*via* the persistent homology of the Vietoris–Rips filtration based on these shortest-path lengths) and geometrically [*via* a Pearson correlation (Pearson, 1895) with some given base-geometry], when possible. Furthermore, we analyze the point cloud given by the columns (or, equivalently, rows) of *D*_iso_, that is, we analyze the entries in *p*_dist_(*D*_iso_).

Note that a contagion map with threshold *T* = 0 is approximately equivalent to a version of Isomap that uses an unweighted neighborhood graph.

### 2.3. Workflow

**Figure 1:**
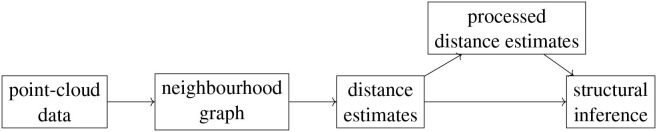
Schematic representation of the workflow.

Our workflow is composed of multiple stages, at each of which one can choose from a number of different options. This leads to exponentially many possible procedures that one can use to analyze a given data set. First, given point-cloud data, one needs to choose the type of neighborhood graph to build on this data set as well as the defining parameter *k* or ϵ. Next, one needs to pick a way of estimating geodesic distances based on this graph. We choose either shortest-path distances or activation times in a threshold contagion, that is, we follow either the Isomap algorithm or that of contagion maps. In the case of contagion maps, one also needs to choose a threshold parameter *T*. Given the set of estimates for the geodesic distances, i.e., the dissimilarity matrix that encodes the shortest-path (*D*_iso_) distances or activation times (*D*_cont_), one can apply further methods either to these estimates directly or to the pairwise distances between the points whose coordinate vectors are the columns (or rows, by symmetry) of this dissimilarity matrix. For either of these choices one can finally apply methods to determine dimensionality, topology, and geometry. Figure 1 shows a schematic representation of our workflow. We apply different subsets of the full analysis to the different data sets that we study.

## 3. Results

We apply Isomap, as well as contagion maps with several different thresholds, to three different data sets. First, we consider point samples from a Swiss roll, a classical benchmark data set for dimensionality reduction and one of the first data sets to which Isomap was applied (Tenenbaum et al., 2000). We then analyze a couple of representatives of the class of torus-based networks that was studied in Mahler (2021). The toroidal structure underlying these networks allows us to examine the topological aspect of our methods by looking to recover the torus' non-trivial topological features. Finally, we consider a data set that represents the conformation space of the cyclo-octane molecule (Martin et al., 2010). This data set is known to have non-trivial topological features and is an example of a naturally occurring data set.

### 3.1. Noisy Samples From a Swiss Roll

We first examine point samples from a Swiss roll that are obtained by taking regularly spaced points on the Swiss roll surface and then adding various levels of noise to these points. This way of generating data makes it possible to have direct control over the data, so one can explore how different algorithms react to slight variations of the data (in terms of e.g., density, noise level, or uniformity). We use this data set primarily to explore the effects of the parameter *k* or ϵ when building a neighborhood graph.

**Figure 2:**
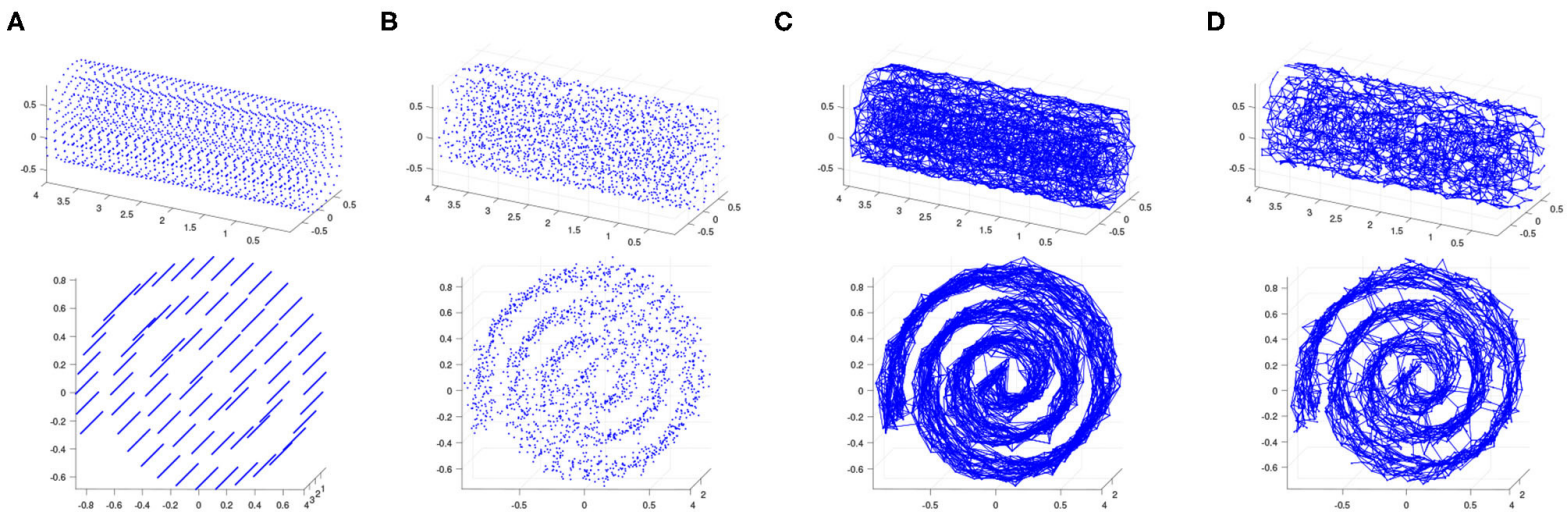
**(A)** Regularly spaced points on a Swiss roll at density 50 per unit area. **(B)** Gaussian noise is added at a signal-to-noise ratio of 30. **(C)** The 5-nearest-neighbor graph on the noisy point cloud. **(D)** The 0.18-neighborhood graph on the noisy point cloud. We show two views of each plot.

We start by taking points on a Swiss roll at a density of approximately 50 per unit area, regularly spaced with respect to the intrinsic geodesic distance on the Swiss roll (see Figure 2A). We then add *Gaussian noise* with a specified signal-to-noise ratio (*S*/*N*) to these regularly spaced points (see Figure 2B). That is, for each point *p* = [*p*_1_, *p*_2_, *p*_3_] in this regularly spaced point sample, we add independent, identically distributed noise drawn from a zero-mean normal distribution to each of its coordinates to obtain a perturbation *p*_noisy_ of the point:


$$p_{\text{noisy}}=\left[p_{1}+n_{1},p_{2}+n_{2},p_{3}+n_{3}\right],$$

where $n_{i}\sim N\left(0,\sigma^{2}\right)$ with $\sigma^{2}=10^{\frac{-S/N}{10}}$.

We test Isomap and contagion maps on this noisy point cloud to see how well each of them sees the underlying 2-dimensional space. Each algorithm starts by building a neighborhood graph on the points (see Figures 2C,D).

We have already touched on Isomap's sensitivity to “short-circuit errors” in the introduction. However, a careful choice of ϵ (or *k*) when constructing the neighborhood graph can mitigate such errors to some extent (Balasubramanian et al., 2002). The goal is to find an ϵ (or *k*) that is small enough to avoid short-circuit edges but not so small that the resulting graph “corrupts” the underlying space. One may choose to simply vary ϵ over a range and implement Isomap on all of the neighborhood graphs that thus arise. This approach is in the same vein as considering the full range of thresholds for the contagion map algorithm, and it works whenever there exists a range of ϵ (or *k*) for which the resulting neighborhood graphs correctly represent the underlying topology. However, for some data sets—particularly those that are sparse and incorporate a high level of noise—it is impossible to find a value for ϵ (or *k*) that strikes a balance between covering the underlying topology and not making “short-circuit errors”. In other words, the range of ϵ (or *k*) that “corrupt” the underlying topology and the range of ϵ (or *k*) that make “short-circuit errors” overlap, leaving no values of ϵ (or *k*) that yield neighborhood graphs that correctly represent the underlying topology. For such data sets, Isomap is inadequate as a manifold-learning tool, but contagion maps may be effective.

**Figure 3:**
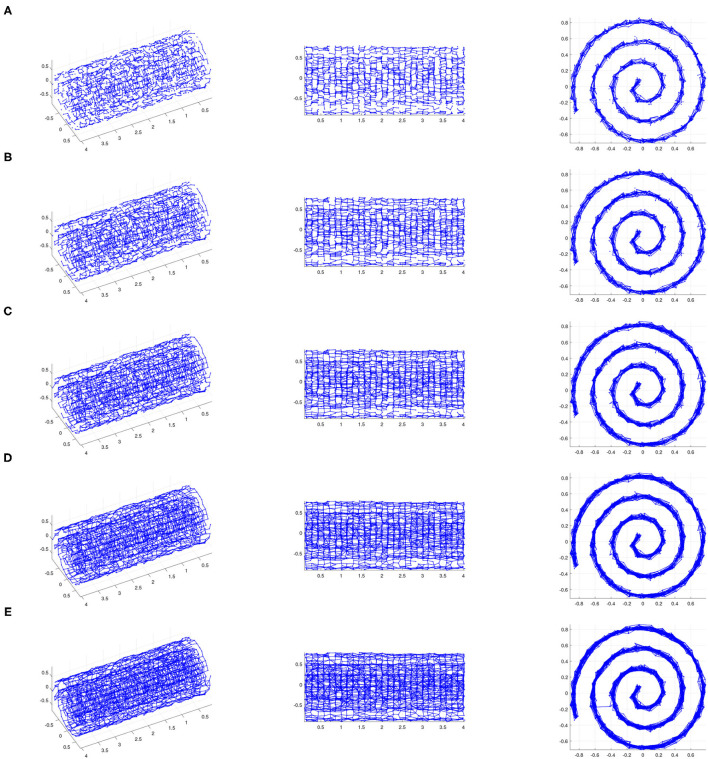
The ϵ-neighborhood graphs on a noisy point sample from a Swiss roll with *S*/*N* = 35 for **(A)** ϵ = 0.14, **(B)** ϵ = 0.15, **(C)** ϵ = 0.16, **(D)** ϵ = 0.17, and **(E)** ϵ = 0.18 (We show three views of each plot.). If ϵ is too small (e.g., ϵ = 0.14), the ϵ-neighborhood graph does not adequately “cover” the underlying Swiss roll. If ϵ is too large (e.g., ϵ = 0.18), the ϵ-neighborhood graph includes inter-sheet edges, and thus does not represent the underlying Swiss roll. However, there exists a range of ϵ for which the ϵ-neighborhood graph covers the underlying surface and does not include inter-sheet edges, thus providing an authentic representation of the underlying Swiss roll. In other words, the pairwise distances between points whose corresponding nodes are adjacent in the ϵ-neighborhood graph for such ϵ approximate the actual geodesic sufficiently, and approximate pairwise geodesic distances between other point pairs can be inferred through the Isomap algorithm. This is an example of a data set for which Isomap is suitable as a manifold-learning technique with a careful choice of ϵ.

**Figure 4:**
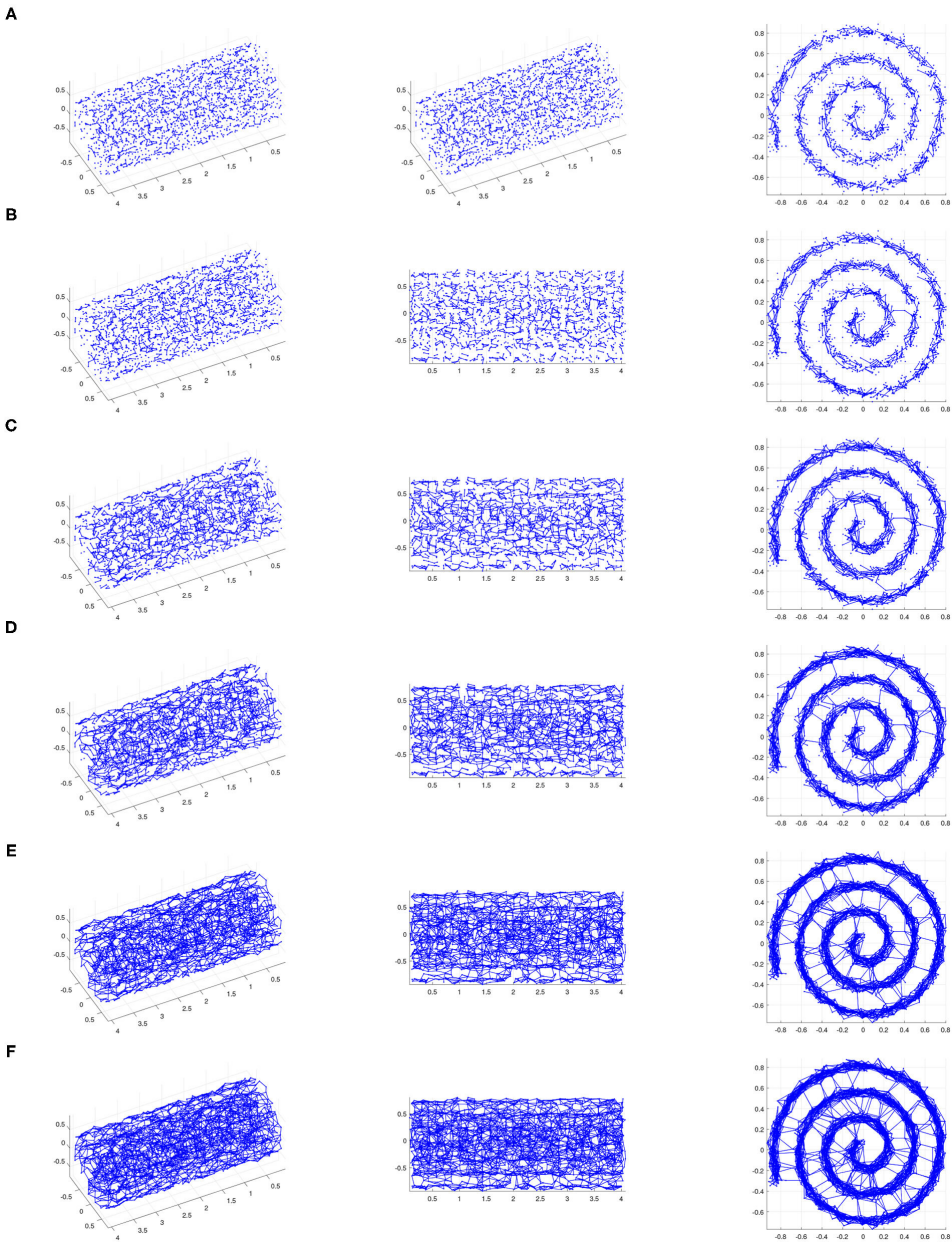
The ϵ-neighborhood graphs on a noisy point sample from a Swiss roll with *S*/*N* = 30 for **(A)** ϵ = 0.11, **(B)** ϵ = 0.12, **(C)** ϵ = 0.14, **(D)** ϵ = 0.16, **(E)** ϵ = 0.18, and **(F)** ϵ = 0.19 (We show three views of each plot.). For small ϵ, the ϵ-neighborhood graph does not adequately “cover” the underlying Swiss roll. As ϵ increases, noisy inter-sheet edges appear (for e.g., ϵ = 0.12) before ϵ is large enough for the neighborhood graph to adequately “cover” the underlying Swiss roll. This is an example of a data set for which Isomap cannot be used successfully as a manifold-learning technique with any choice of ϵ.

**Figure 5:**
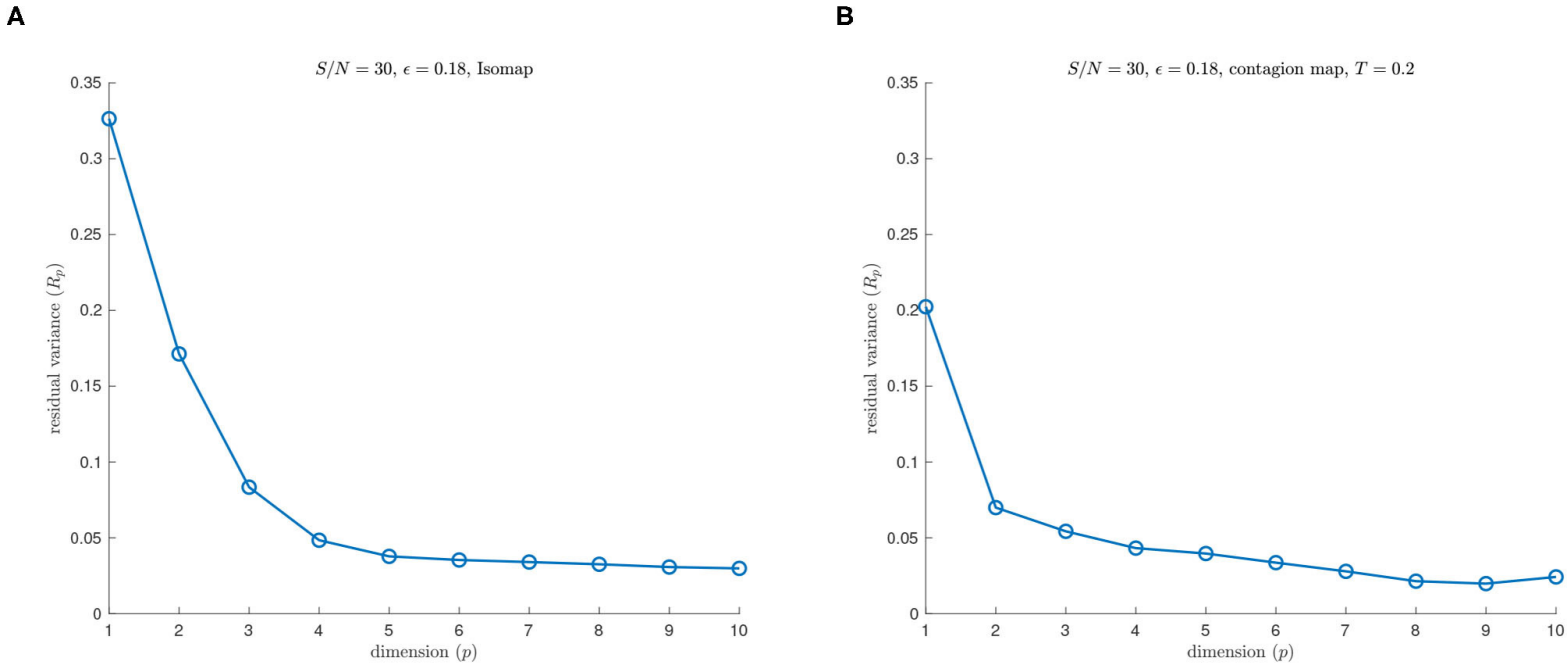
**(A)** Isomap and **(B)** contagion map with *T* = 0.2 (both starting with a 0.18-neighborhood graph) on a noisy sample from a Swiss roll with *S*/*N* = 30.

See Figures 3, 4 for examples on the Swiss roll that illustrate the concepts in the above paragraph. In particular, Figure 3 shows an example of a data set for which a careful choice of ϵ generates a neighborhood graph that both captures the underlying manifold and does not include “short-circuit” edges. By contrast, Figure 4 shows an example of a data set for which no choice of ϵ produces a neighborhood graph that accurately represents the underlying manifold. Figure 5 shows the residual variances of projecting this data set *via* MDS to dimensions 1 to 10 when based on Isomap and when based on the contagion map with *T* = 0.2 (both starting with a 0.18-neighborhood graph). For the contagion map, the residual variance plunges at dimension 2, thereby correctly identifying the intrinsic dimension of the data. The residual variances for Isomap, on the other hand, only decrease slighty and continuously across the increasing target dimensions. That is, Isomap fails to see the correct intrinsic structure of the data when starting with an ϵ-neighborhood graph with ϵ = 0.18 (or any other value of ϵ), while contagion map — with a suitable choice of ϵ and *T* — correctly identifies the underlying structure. For contagion maps to work in this case, the value of ϵ had to be chosen large enough for the neighborhood graph to cover the underlying manifold, and the value of *T* had to be picked small enough to carry the contagion and large enough to be resistant to the unavoidable noisy inter-sheet edges in the ϵ-neighborhood graph.

We now consider 2,000 points of the Swiss roll data set^4^ from Tenenbaum et al. (2000). This data set consists of 20,000 points in total.

**Figure 6:**
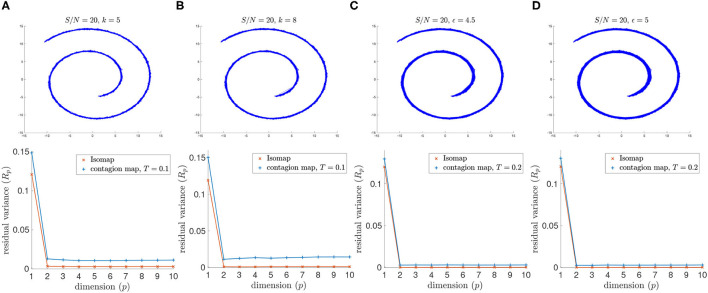
(Top row) Neighborhood graphs on 2,000 points of the Swiss roll data set from Tenenbaum et al. (2000) with white Gaussian noise added with *S*/*N* = 20. (Bottom row) The residual variances of MDS projections to dimensions 1–10 resulting from the approximate pairwise geodesic distances between nodes based on shortest paths on the weighted graph (i.e., Isomap) and the activation times in a threshold contagion on the unweighted graph (i.e., contagion maps). The neighborhood graphs are **(A)** the 5-nearest-neighbor graph, **(B)** the 8-nearest-neighbor graph, **(C)** the 4.5-neighborhood graph, and **(D)** the 5-neighborhood graph.

Both Isomap and contagion maps perform adequately for the high signal-to-noise ration of *S*/*N* = 20 (i.e., low noise level) with all four examined versions of neighborhood graphs (see Figure 6).

**Figure 7:**
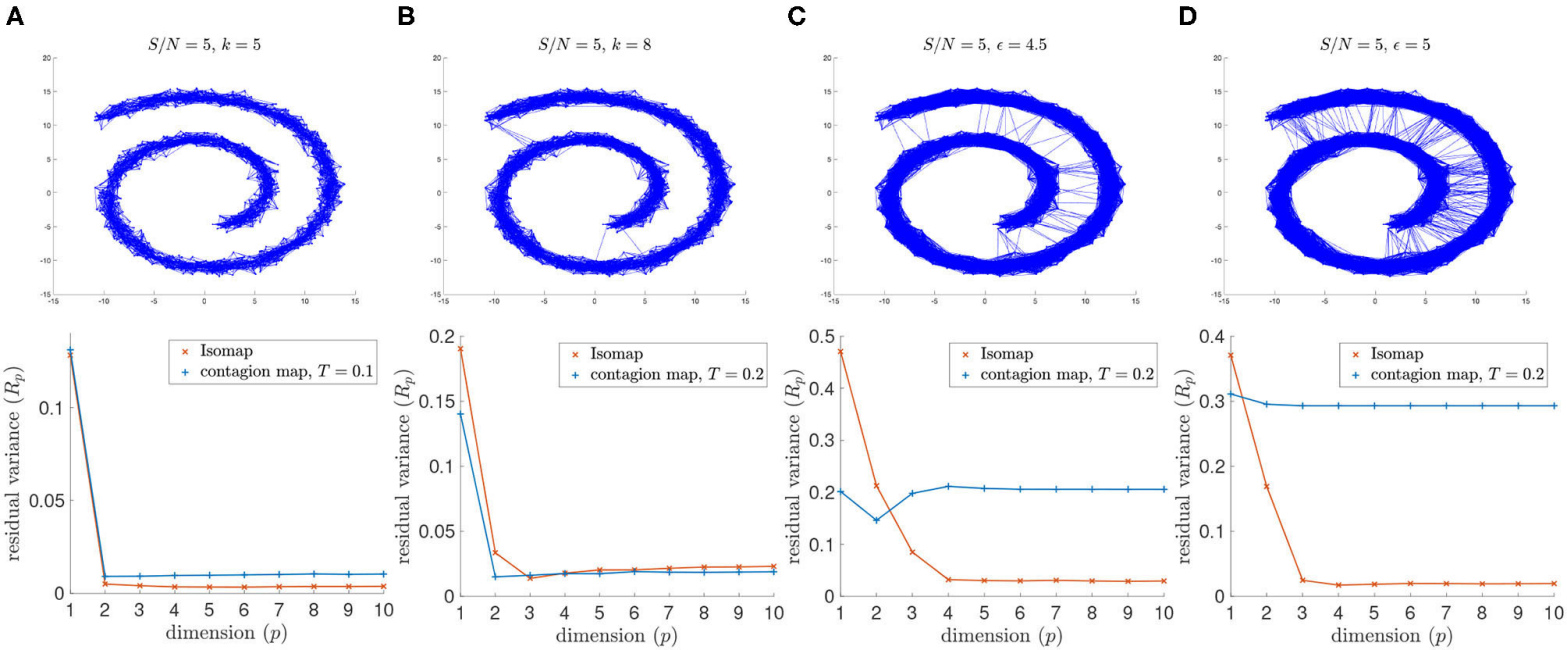
(Top row) Neighborhood graphs on 2,000 points of the Swiss roll data set from Tenenbaum et al. (2000) with white Gaussian noise added with *S*/*N* = 5. (Bottom row) The residual variances of MDS projections to dimensions 1 to 10 resulting from the approximate pairwise geodesic distances between nodes based on shortest paths on the weighted graph (i.e., Isomap) and the activation times in a threshold contagion on the unweighted graph (i.e., contagion maps). The neighborhood graphs are **(A)** the 5-nearest neighbor graph, **(B)** the 8-nearest neighbor graph, **(C)** the 4.5-neighborhood graph, and **(D)** the 5-neighborhood graph.

For the lowest signal-to-noise ratio (i.e., greatest noise level) that we consider (namely *S*/*N* = 5) the 8-nearest-neighbor graph includes noisy inter-sheet edges. As a result, Isomap does not detect the intrinsic dimension 2 of the Swiss roll when using the 8-nearest neighbor graph, and neither does the contagion map with *T* = 0 or *T* = 0.1, thresholds for which the activation times in our threshold contagion are close to the shortest paths in the unweighted neighborhood graph (see Figure 7). With *T* = 0.2, however, the contagion map does correctly recover the intrinsic dimension 2, as this threshold is just large enough to be robust to the occurring noisy edges. For thresholds larger than *T* = 0.2, many of the realizations of our threshold contagion leave nodes in the neighborhood graph inactive (recorded as “infinite” activation times), and, as a result, the residual variances of the embeddings based on these activation times are large for all considered dimensions (1 to 10). This illustrates that, while contagion maps can be a powerful tool when dealing with such noisy edges, a suitable choice of threshold can be a delicate matter.

**Figure 8:**
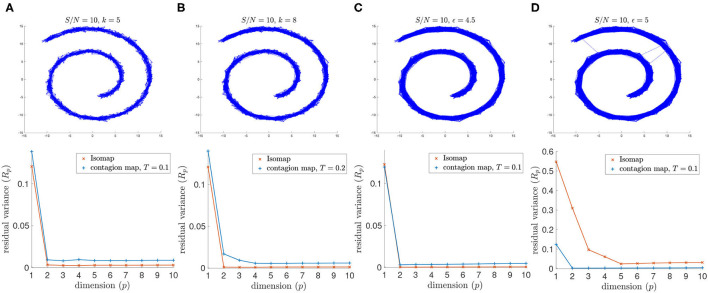
(Top row) Neighborhood graphs on 2,000 points of the Swiss roll data set from Tenenbaum et al. (2000) with white Gaussian noise added with *S*/*N* = 10. (Bottom row) The residual variances of MDS projections to dimensions 1 to 10 resulting from the approximate pairwise geodesic distances between nodes based on shortest paths on the weighted graph (i.e., Isomap) and the activation times in a threshold contagion on the unweighted graph (i.e., contagion maps). The neighborhood graphs are **(A)** the 5-nearest neighbor graph, **(B)** the 8-nearest neighbor graph, **(C)** the 4.5-neighborhood graph, and **(D)** the 5-neighborhood graph.

Similarly, for signal-to-noise ratio *S*/*N* = 5, Isomap fails when based on the 4.5-neighborhood graph or the 5-neighborhood graph, but the contagion map for a threshold of *T* = 0.2 successfully identifies the intrinsic dimension 2 for both examined values of the neighborhood parameter ϵ. Furthermore, when based on a 5-neighborhood graph, Isomap fails even for the higher *S*/*N* = 10, as the 5-neighborhood graph includes noisy inter-sheet edges even for this lower noise level (see Figure 8).

Note that our measures for topology and geometry are not useful for this data set, as the underlying manifold has no non-trivial topological features, and there is no base-geometry provided.

Our experiments on this classical manifold-learning data showcase examples where contagion maps have the power to detect low-dimensional structure, even when Isomap is unsuccessful. Crucial to contagion maps' success is the careful choice of suitable neighborhood graph parameter and contagion threshold.

### 3.2. Torus-Based Networks

We consider the torus-based model described in Mahler (2021) with *N* = 2,500 nodes and a degree of *geometric edges* of *d*^G^ = 8. Networks in this model are similar to Kleinberg's small-world like network (Kleinberg, 2000). They consist of a periodic grid of nodes that are connected *via geometric edges* (i.e., edges between neighboring nodes) in a regular manner, and to which *non-geometric edges* are added according to a probability distribution. We add first 2 and then 4 non-geometric edges per node uniformly at random and apply versions of both Isomap and contagion maps (with thresholds *T* = 0, 0.1, …, 1) to the resulting networks. Namely, we calculate the shortest-path lengths between pairs of nodes in these two unweighted networks, as well as the activation times in all realizations of our threshold contagion that are seeded at the direct neighborhoods of individual nodes. We thus obtain two dissimilarity matrices: one holding the shortest-path lengths (*D*_iso_) and one holding the symmetrized activation times (*D*_cont_). We analyze the information held in these two dissimilarity matrices geometrically, topologically, and in terms of dimensionality in two ways each. We first analyze the estimated pairwise geodesic distances held in each dissimilarity matrix directly, and we then consider the point clouds that results from taking columns (or, equivalently, rows) of each dissimilarity matrix as the coordinate vectors of points in ℝ^2500^.

In detail, we perform the following analyzes:

- In terms of dimensionality: We perform MDS based on the entries in each dissimilarity matrix to dimensions 1–10 and record the residual variance for each dimension. We also perform MDS on the point cloud that results from taking columns (or, equivalently, rows) of each dissimilarity matrix as the coordinate vectors of points in ℝ^2500^. In both cases, we identify the approximate embedding dimension as the lowest dimension such that the residual variance when projecting down to that dimension *via* MDS is below 5%.
- Topologically: We build Vietoris–Rips filtrations based on the approximations to the pairwise geodesic distances (i.e., the entries in each dissimilarity matrix) and compute their persistent homologies. We also build Vietoris–Rips filtrations on the points cloud that results from taking columns (or, equivalently, rows) of each dissimilarity matrix as the coordinate vectors of points in ℝ^2500^ and compute their persistent homologies.
- Geometrically: We calculate the Pearson correlation coefficient between the entries in each dissimilarity matrix and the corresponding pairwise distances between regularly spaced points on a torus:
  (1) $$\begin{array}{r} \begin{array}{c} \frac{1}{2\pi }\left(\cos\frac{2\pi x}{n},\sin\frac{2\pi x}{n},\cos\frac{2\pi y}{n},\sin\frac{2\pi y}{n}\right)\in \\ 𝕋=\frac{1}{2\pi }{𝕊}^{1}\times \frac{1}{2\pi }{𝕊}^{1}\subset {\mathbb{R}}^{4},\\ x,y\in \left\{0,1,\ldots ,n-1\right\}. \end{array} \end{array}$$
  We also calculate the Pearson correlation coefficient between the pairwise distances between points in the point cloud that results from taking columns (or, equivalently, rows) of each dissimilarity matrix as the coordinate vectors of points in ℝ^2500^ and the corresponding pairwise distances between the regularly spaced points on a torus specified in (1).

We find that Isomap is unable to infer the underlying torus structure from these networks. Contagion maps, however, detect the characteristics of the torus when using a threshold of *T* ≈ 0.2. The results in this section illustrate the utility of contagion maps for spatial network data that incorporates noisy edges and its potential to outperform Isomap in such scenarios, but they also highlight that a careful choice of *T* is critical.

#### Dimensionality

**Figure 9:**
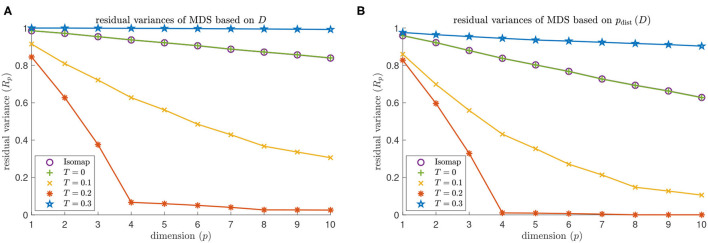
Dimensionality results on our torus-based network with *d*^NG^ = 2. **(A)** Residual variances of MDS based on the estimated geodesic distances (i.e., the entries in *D*_iso_ and *D*_cont_) according to Isomap, and according to contagion maps with thresholds *T* = 0, *T* = 0.1, *T* = 0.2, and *T* = 0.3. **(B)** Residual variances of MDS based on the point cloud (i.e., the rows of the *D*_iso_ and *D*_cont_) according to Isomap, and according to contagion maps with thresholds *T* = 0, *T* = 0.1, *T* = 0.2, and *T* = 0.3.

**Figure 10:**
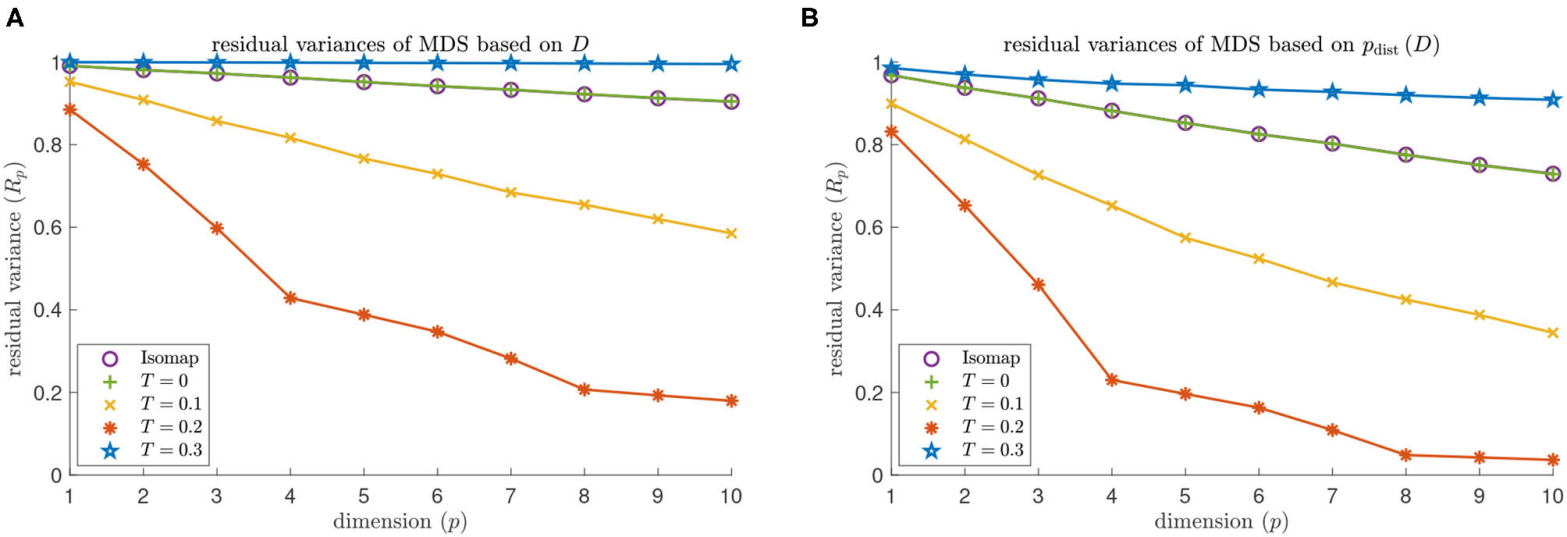
Dimensionality results on our torus-based network with *d*^NG^ = 4. **(A)** Residual variances of MDS based on the estimated geodesic distances (i.e., the entries in *D*_iso_ and *D*_cont_) according to Isomap, and according to contagion maps with thresholds *T* = 0, *T* = 0.1, *T* = 0.2, and *T* = 0.3. **(B)** Residual variances of MDS based on the point cloud (i.e., the rows of the *D*_iso_ and *D*_cont_) according to Isomap, and according to contagion maps with thresholds *T* = 0, *T* = 0.1, *T* = 0.2, and *T* = 0.3.

MDS based on Isomap does not identify the embedding dimension 4 of a torus for either *d*^NG^ = 2 or *d*^NG^ = 4 (see the purple data points in Figures 9, 10). The residual variance based on contagion maps does have a dip at dimension 4 for the threshold *T* = 0.2 (see the red data points in Figures 9, 10) but does not when the threshold is *T* = 0.1 or *T* = 0.3. Note that for Isomap, as well as contagion maps with all considered thresholds, the residual variances are smaller for all considered dimensions when the analysis is done on the point cloud, making the results for contagion map with *T* = 0.2 look sharper.

**Figure 11:**
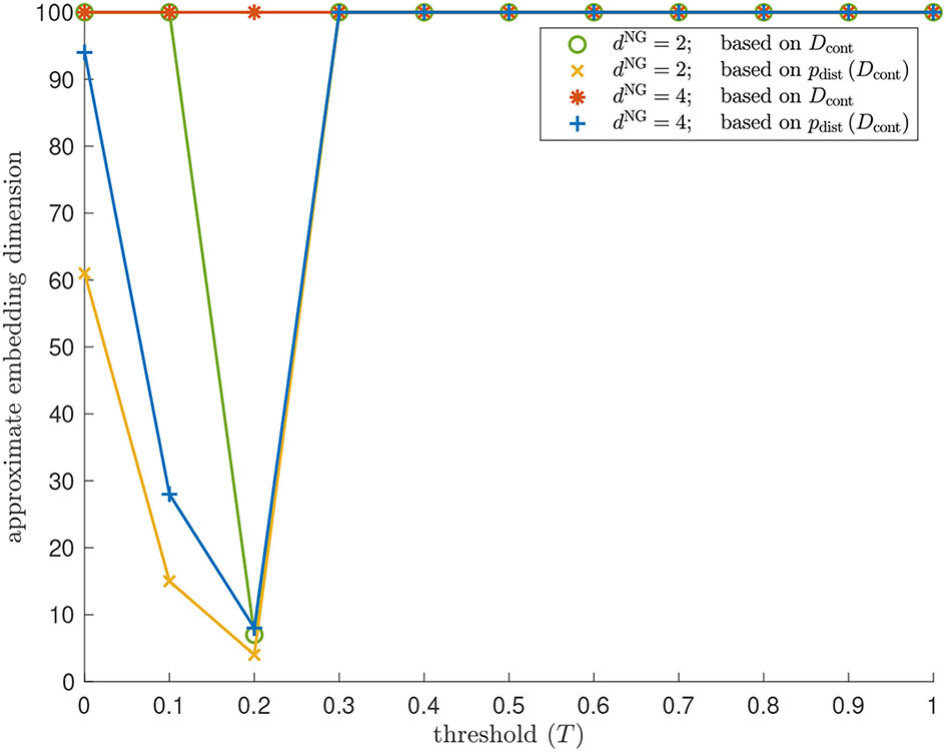
Dimensionality results on our torus-based network with *d*^NG^ = 2 (colored in green and yellow) and *d*^NG^ = 4 (colored in red and blue). Approximate embedding dimension according to contagion maps with different values of threshold *T* and critical value 5% using the dissimilarity matrix *D*_cont_ (colored in green and red), and the point cloud whose coordinate vectors are the rows in *D*_cont_ (colored in yellow and blue). For *d*^NG^ = 2, the results for Isomap are practically identical to those for the contagion map with *T* = 0: The approximate embedding dimension is at least 100 (which is the dimension at which we cap our computations) when based on the entries in *D*_iso_, and it is 61 when based on the point cloud whose coordinate vectors are given by the rows of *D*_iso_. The approximate embedding dimension according to contagion maps reaches a minimum value for *T* = 0.2 both when working with *D*_cont_ and when working with *p*_dist_(*D*_cont_). This minimal value is 7 in the former case (see the data points colored in green), and is 4 in the latter case (see the data points colored in yellow). Similarly, for *d*^NG^ = 4, the results for Isomap are practically identical to those for the contagion map with *T* = 0: The approximate embedding dimension is at least 100 (which is the dimension at which we cap out computations) when based on the entries in *D*_iso_; and it is 95 when based on the point cloud whose coordinate vectors are given by the rows of *D*_iso_. The approximate embedding dimension according to contagion maps reaches a minimum value of 8 for *T* = 0.2 when working with *p*_dist_(*D*_cont_) (see the data points colored in blue).

**Figure 12:**
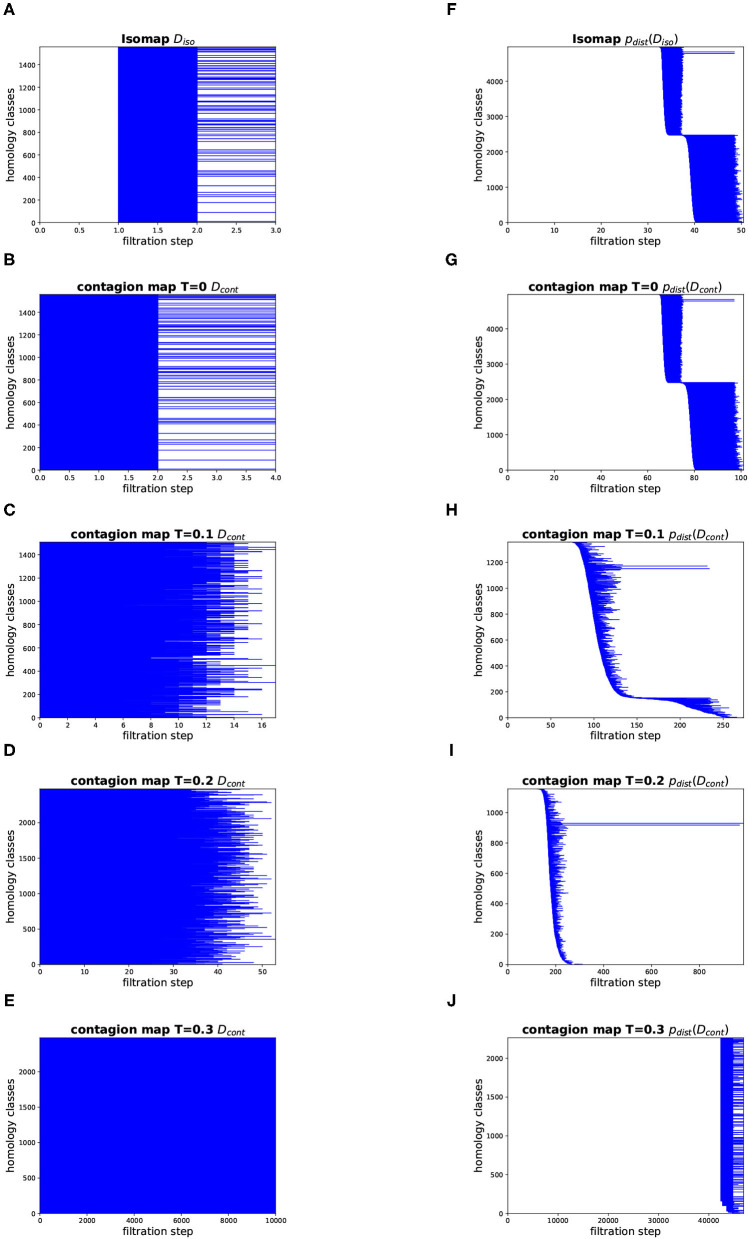
Topology results on our torus-based network with *d*^NG^ = 2. (Left column) Dimension 1 barcodes of the Vietoris–Rips filtrations based on the estimated geodesic distances (i.e., the entries in *D*_iso_ and *D*_cont_) according to **(A)** Isomap, and according to contagion maps with **(B)** *T* = 0, **(C)** *T* = 0.1, **(D)** *T* = 0.2, and **(E)** *T* = 0.3. (Right column) Barcodes of the Vietoris–Rips filtrations on the point clouds according to **(F)** Isomap, and according to contagion maps with **(G)** *T* = 0, **(H)** *T* = 0.1, **(I)** *T* = 0.2, and **(J)** *T* = 0.3.

We identify the approximate embedding dimensions for Isomap and contagion maps with thresholds *T* = 0,0.1, … ,1 and show the results in Figure 11. Both versions of Isomap (the one performing MDS based on the entries in *D*_iso_ and the one performing MDS based on the distances between the rows of *D*_iso_) vastly overestimate the embedding dimension for the torus-based network with *d*^NG^ = 2 and the one with *d*^NG^ = 4. When performing MDS based on the entries in *D*_iso_, the approximate embedding dimension is at least 100 (which is the dimension at which we cap our computations). When performing MDS based on the distances between the rows of *D*_iso_, the embedding dimensions are still very large: The embedding dimension is 61 for the network with *d*^NG^ = 2, and it is 95 for the network with *d*^NG^ = 4. Note that these results are practically identical to those the for the contagion map with *T* = 0, as we are working with the same unweighted graphs in both Isomap and contagion maps. For contagion maps with varying thresholds *T*, the approximate embedding dimension has a dip around *T* = 0.2, except when performing MDS based on the entries in *D*_cont_ for the network with *d*^NG^ = 4, in which case contagion maps return an embedding dimension of at least 100 for all thresholds (see the red data points in Figure 12). This suggests that, for thresholds close to 0.2, the contagion spreads predominantly *via* WFP along the underlying torus, making it possible to identify the underlying low-dimensional structure.

#### Topology

**Figure 13:**
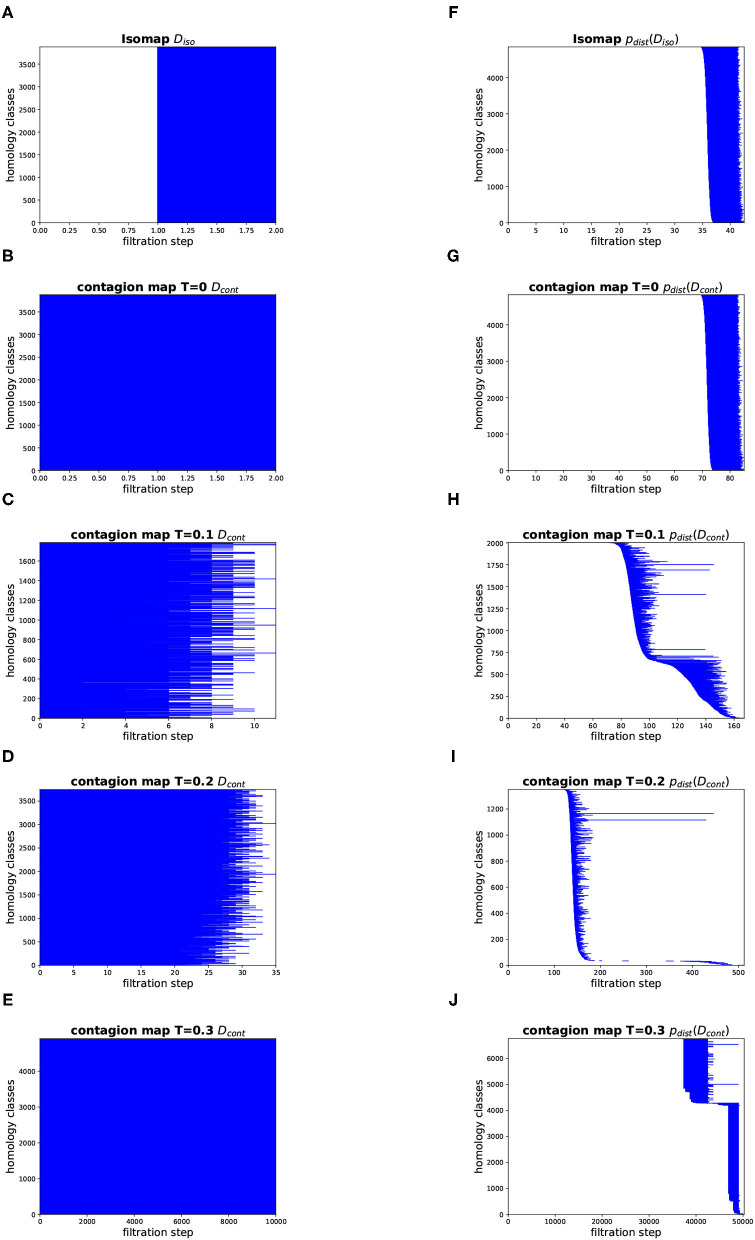
Topology results on our torus-based network with *d*^NG^ = 4. (Left column) Dimension 1 barcodes of the Vietoris–Rips filtrations based on the estimated geodesic distances (i.e., the entries in *D*_iso_ and *D*_cont_) according to **(A)** Isomap, and according to contagion maps with **(B)** *T* = 0, **(C)** *T* = 0.1, **(D)** *T* = 0.2, and **(E)** *T* = 0.3. (Right column) Barcodes of the Vietoris–Rips filtrations on the point clouds according to **(F)** Isomap, and according to contagion maps with **(G)** *T* = 0, **(H)** *T* = 0.1, **(I)** *T* = 0.2, and **(J)** *T* = 0.3.

Figures 12, 13 show the barcodes corresponding to the persistent homology in dimension 1 of the Vietoris–Rips filtrations built according to the different versions of Isomap and contagion maps. The barcodes in dimension 1 of the Vietoris–Rips filtrations based on the estimated geodesic distances (i.e., the entries in *D*_iso_ and *D*_cont_) do not seem to reveal any significant features for either Isomap or contagion maps (see Figures 12A–E, 13A–E). The barcode in dimension 1 of the Vietoris–Rips filtration on the point cloud based on Isomap (i.e., given by the rows of *D*_iso_) on the network with *d*^NG^ = 2 does feature two dominant bars (see Figure 12F), as do the barcodes corresponding to point clouds based on contagion maps for *T* = 0, *T* = 0.1, and *T* = 0.2 (i.e., given by the rows of *D*_cont_) (see Figures 12G–I). Note, however, that in panels F–H, due to the order of the bars, the dominance appears slightly stronger than it actually is. For the network with *d*^NG^ = 4, the barcode in dimension 1 of the Vietoris–Rips filtration on the point cloud based on Isomap does not have any dominant bars, whereas the one based on the contagion map for *T* = 0.2 does (see Figure 13I).

#### Geometry

**Figure 14:**
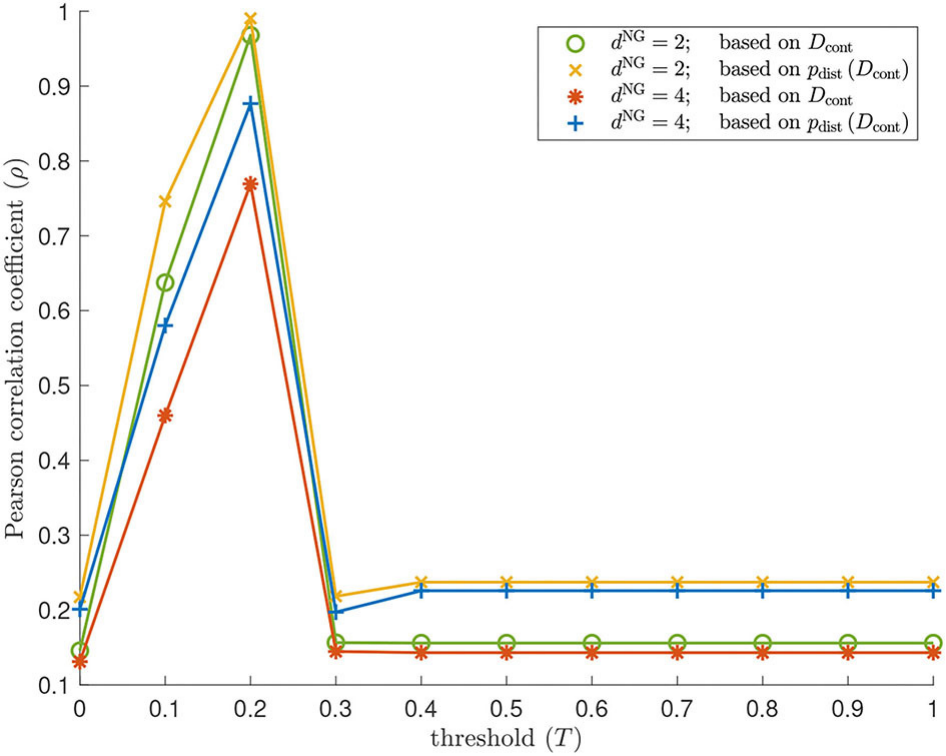
Geometry results on our torus-based network with *d*^NG^ = 2 and *d*^NG^ = 4. Pearson correlation coefficient between the pairwise distances between the regularly spaced points on a torus specified in (1) and the following sets: the estimated geodesic distances (i.e., the entries in *D*_cont_) according to contagion maps with thresholds *T* = 0, 0.1, …, 1 on a torus-based network with *d*^NG^ = 2 (colored in green); the pairwise distances between points in ℝ^2500^ whose coordinate vectors are the rows of *D*_cont_ according to contagion maps with thresholds *T* = 0, 0.1, …, 1 on a torus-based network with *d*^NG^ = 2 (colored in yellow); the estimated geodesic distances (i.e., the entries in *D*_cont_) according to contagion maps with thresholds *T* = 0, 0.1, …, 1 on a torus-based network with *d*^NG^ = 4 (colored in red); the pairwise distances between points in ℝ^2500^ whose coordinate vectors are the rows of *D*_cont_ according to contagion maps with thresholds *T* = 0, 0.1, …, 1 on a torus-based network with *d*^NG^ = 4 (colored in blue). The results for Isomap are practically identical to those for the contagion map with *T* = 0: The Pearson correlation coefficient is 0.1458 (0.1311) when based on the entries in *D*_iso_ for a torus-based network with *d*^NG^ = 2 (*d*^NG^ = 4), and it is 0.2175 (0.2013) when based on the point cloud whose coordinate vectors are given by the rows of *D*_iso_ for a torus-based network with *d*^NG^ = 2 (*d*^NG^ = 4).

We examine the geometry of our Isomap and contagion map results by comparing the entries of *D*_iso_ and *D*_cont_, as well as the point clouds given by the rows of these dissimilarity matrices, to the regularly spaced points on a torus specified in (1) *via* the Pearson correlation coefficient (see Figure 14). Isomap returns a low Pearson correlation in all cases, suggesting that the shortest-path distances are (as expected, given the large number of non-geometric edges) not good estimates for the distances along the torus that underlies these networks. Note that these results are practically identical to those for contagion map with *T* = 0, as we are working with the same unweighted graphs in both Isomap and contagion maps. For contagion maps with varying thresholds *T*, the Pearson correlation coefficient peaks around *T* = 0.2, suggesting that, for thresholds close to *T* = 0.2, the contagion spreads predominantly *via* WFP, making the activation times good estimates for the distance along the torus that underlies these networks.

These synthetic torus-based network data sets allowed us to explore all three structural measures (dimensionality, topology, and geometry), as the data's underlying structure is not only low-dimensional but has non-trivial topological features to recover, and we have a base-geometry to compare our contagion and shortest-path based distance estimates against.

Again, we emphasized the importance of finding a suitable value for the contagion threshold. In the case of these torus-based network data sets, a range of thresholds around *T* = 0.2 produce contagion maps that reveal the underlying toroidal structure. This optimal range for *T* could be predicted from the bifuracation analysis in Mahler (2021). In a true manifold-learning application, however, where we have no a priori knowledge about the data's underlying structure and how it is sampled from that underlying space, it is generally difficult to guess an appropriate value of *T*. A practical approach is to simply sweep over a range of incremental values of *T* and look for dips and peaks in the measures for topology, geometry, and dimensionality. Such dips and peaks correspond to values of *T* for which the contagion spreads predominantly as a wavefront, and therefore yields activation times that give good estimates for the actual intrinsic distances between data points and allow recovery of the underlying structure.

We also demonstrated that processing our distance estimates through *p*_dist_ before performing our analyzes to determine dimensionality, topology, and geometry improves these measures, giving clearer results. In some cases we saw that *p*_dist_ appears, in fact, to be a crucial step in the manifold-learning pipeline.

### 3.3. Conformation Space of the Cyclo-Octane Molecule

The cyclo-octane molecule (*CH*_2_)_8_ consists of a ring of eight carbon atoms, each bonded with two hydrogen atoms. A *conformation* of a molecule is a possible spatial arrangement of its atoms (modulo rotation and translation) (Moss, 1996). The conformation of a molecule can be specified by the coordinates of each of its atoms in three-dimensional space, giving a point in ℝ^3*a*^, where *a* is the number of atoms in the molecule. (In this case, each coordinate of each atom in three-dimensional space is a feature and ℝ^3*a*^ is the feature space). The set of such points for all conformations of a molecule is called its *conformation space*. Each conformation is accompanied by a state of potential energy of the molecule, and a conformation is more likely to occur the lower its associated potential energy. The cyclo-octane molecule has many conformations of comparable potential energy, and its conformation space has been studied in computational chemistry for over 50 years (Hendrickson, 1967; Pakes et al., 1981). Given the locations of the eight carbon atoms in a conformation of the cyclo-octane molecule, the locations of the hydrogen atoms are determined to minimize energy: The two covalent hydrogen atoms of each carbon atom are positioned to form a tetrahedral arrangement with the two neighboring carbon atoms that minimizes the potential energy of that subunit of the molecule. The conformation space of cyclo-octane thus lies in ℝ^3×8^ = ℝ^24^. It is generally assumed that conformation spaces form low-dimensional manifolds, so identifying the structure of the conformation space of a molecule is essentially a manifold-learning problem. The conformation space of cyclo-octane has been shown to be the union of a sphere with a Klein bottle intersecting in two circles of singularities (Brown et al., 2008; Martin et al., 2010), forming a two-dimensional manifold with singularities.

Martin et al. (2010) analyzed a data set of 6, 040 points in the conformation space of cyclo-octane, subsampled from a larger data set consisting of 1031644 cyclo-octane conformations. This data set is publicly available as part of the javaPlex software package^5^ (Tausz et al., 2014). To visualize this set of points, Martin et al. mapped the points from ℝ^24^ to ℝ^3^ *via* Isomap. We explore different versions of both Isomap and contagion maps on it.

**Figure 15:**
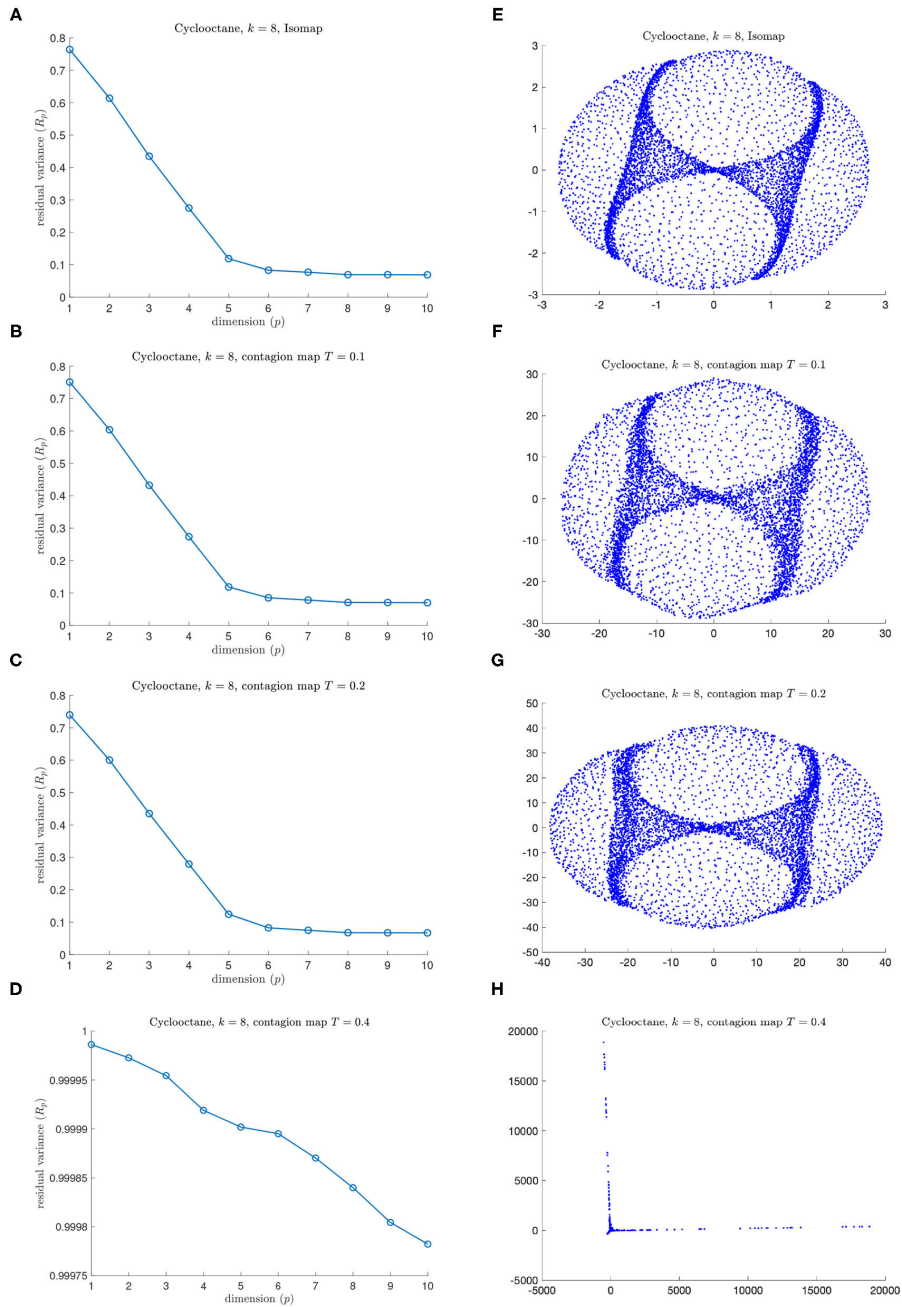
Results for **(A,E)** Isomap and for **(B,C,D,F,G,H)** contagion maps for different values of *T* on the data set of 6040 points in the conformation space of cyclo-octane (using an 8-nearest neighbor neighborhood graph). **(A)** Residual variances for projections *via* MDS onto dimensions 1 to 10 in the original Isomap algorithm. **(E)** Visualization of Isomap in 3D **(B–D)**. Residual variances for projections onto dimensions 1 to 10 in the contagion-map algorithm for **(B)** *T* = 0.1, **(C)** *T* = 0.2, and **(D)** *T* = 0.4 **(F–H)**. Visualizations of projections to 3D for **(F)** *T* = 0.1, **(G)** *T* = 0.2, and **(H)** *T* = 0.4.

Figure 15 shows the residual variances for projections *via* MDS onto dimensions 1 to 10 based on shortest-path distances, i.e., based on the entries in *D*_iso_, (panel A) as well as for those based on activation times in contagions with thresholds *T* = 0.1, 0.2, and 0.4, i.e., based on the entries in *D*_cont_, (panel B–D), and it shows the visualizations of the projection to 3D (panels E–H). We see that Isomap and contagion maps with low thresholds (*T* = 0.1 and 0.2) detect the embedding dimension of the underlying space, suggesting the absence of noisy edges in the 8-nearest-neighbor graph on this data set. Contagion maps with higher thresholds (e.g., 0.4) do not seem to reveal any meaningful structure. This is likely due to the contagion stabilizing before much of the graph has been activated, leading to many “infinite” activation times.

**Figure 16:**
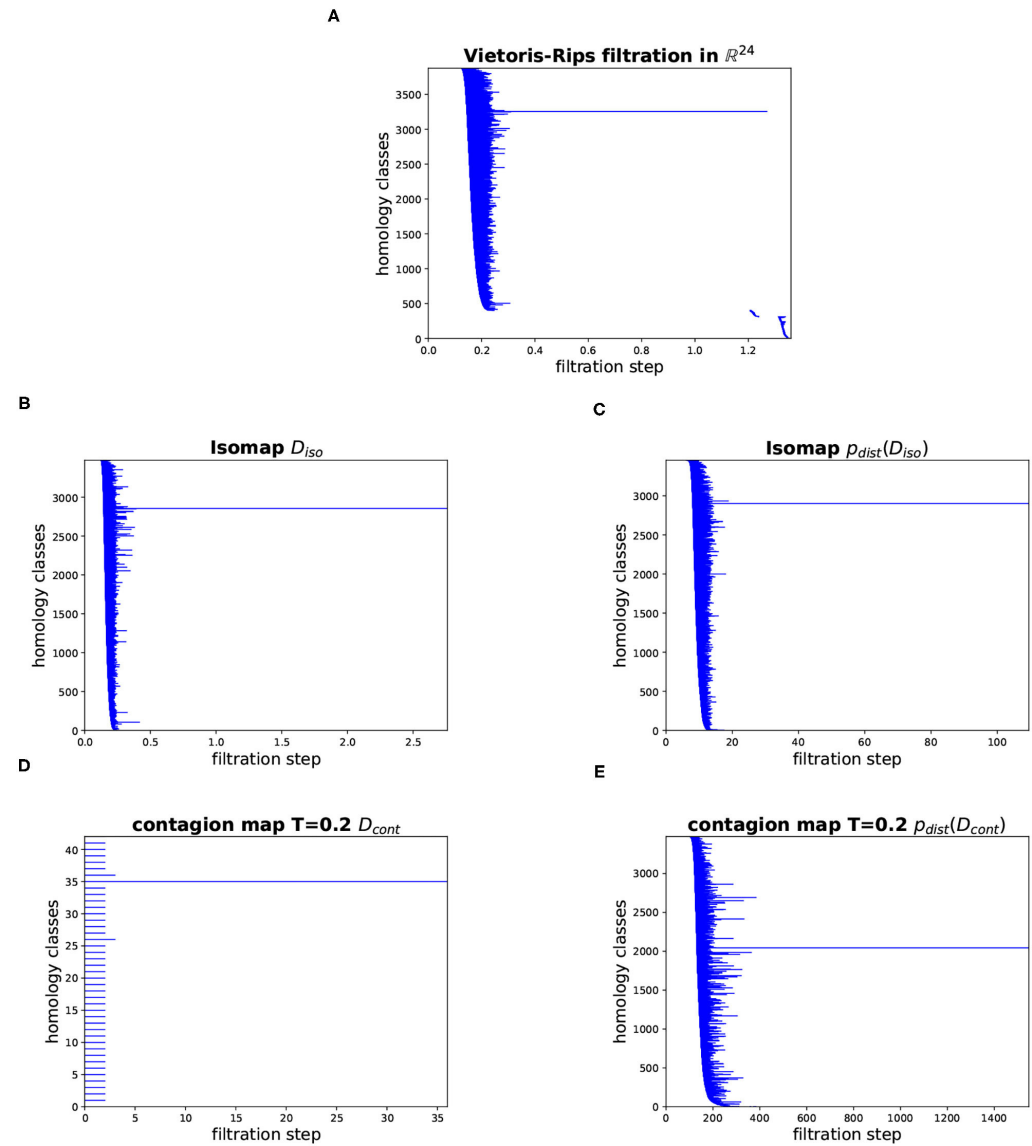
Barcodes for the persistent homology in dimension 1 of various Vietoris–Rips filtrations based on the cyclo-octane data set. **(A)** Vietoris–Rips filtration directly on the data points in ℝ^24^. **(B)** Vietoris–Rips filtration based on the shortest-path distances in the 8-nearest-neighbor graph (i.e., based on the entries in *D*_iso_). **(C)** Vietoris–Rips filtration based on the points whose coordinate vectors are the rows of *D*_iso_ (corresponding to the 8-nearest-neighbor graph). **(D)** Vietoris–Rips filtration based on the activation times of the contagion with threshold *T* = 0.2 on the 8-nearest-neighbor graph (i.e., based on the entries in *D*_cont_). **(E)** Vietoris–Rips filtration based on the points whose coordinate vectors are the rows of *D*_cont_ (corresponding to the contagion with threshold *T* = 0.2 on the 8-nearest-neighbor graph).

In Figure 16, we show barcodes corresponding to the persistent homology in dimension 1 of various Vietoris–Rips filtrations based on the cyclo-octane data set of 6040 points in ℝ^24^. The barcode in Figure 16A corresponds to the Vietoris–Rips filtration built directly on the data points in their ambient space ℝ^24^. This barcode has one dominant bar, suggesting that the Klein bottle and the sphere whose union is the conformation space of cyclo-octane intersect in a way that makes the 1-dimensional loop that is present in the homology of the Klein bottle over ℤ/2ℤ, but not over ℤ, nullhomotopic. The other panels show barcodes corresponding to various Vietoris–Rips filtrations that, in a sense, mimic the Vietoris–Rips filtration built according to the intrinsic metric on the underlying manifold. Namely, they are Vietoris–Rips filtrations based on different versions of Isomap and contagion map, that is, based on estimates for the geodesic distance on the underlying manifold. Figure 16B shows the barcode corresponding to the shortest-path distances in the 8-nearest-neighbor graph (i.e., based on the entries in *D*_iso_). Figure 16C shows the Vietoris–Rips filtration based on the points whose coordinate vectors are the columns of *D*_iso_. Figure 16D shows the Vietoris–Rips filtration based on the activation times of the contagion with threshold *T* = 0.2 on the 8-nearest-neighbor graph (i.e., based on the entries in *D*_cont_). Figure 16E shows the Vietoris–Rips filtration based on the points whose coordinate vectors are the columns of *D*_cont_. All of these barcodes have one dominant bar, which is consistent with the homology of the underlying manifold. The barcode in Figure 16D has many bars with identical birth and death. This stems from the fact that activation times (in our contagion model) have integer values between 0 and 2*N* (where *N* is the number of node or, equivalently, data points), and so a Vietoris–Rips filtration based on these values has only few filtration steps at which simplices are added.

This sample of the conformation space of cyclo-octane is an example of a data set for which Isomap is successful, as are contagion maps with a sufficiently small contagion threshold *T*. Sweeping over a range of values of the threshold *T* includes the value zero, which corresponds to a contagion map that can be seen as equivalent to Isomap. We can therefore view contagion maps as an extension that includes a form of Isomap.

## 4. Conclusion

Isomap is a well-established manifold-learning tool and is useful for many data sets. It can successfully handle curvature of data in many cases, and the freedom of choosing the parameter *k* or ϵ when creating a neighborhood graph allows it to handle noise to some extent. However, when faced with particularly sparse and noisy data, Isomap is prone to so-called “short-circuit errors”, which in some cases cannot be avoided regardless of the choice of *k* or ϵ. For such data, contagion maps can yield better reconstructions. For a suitable choice of the threshold parameter *T*, single noisy edges that occur in a neighborhood graph do not carry a contagion and thus do not distort the estimate of the geodesic distances *via* activation times significantly. In other words, with the right choice of *T*, contagion maps are able to “exploit social reinforcement to silence noise”.

We have demonstrated this on a number of synthetic and real-world data sets, including samples from the Swiss roll, a classical benchmark data set for manifold learning. Some of the data sets we examined were point clouds, on which we built different neighborhood graphs in the first step of our algorithm. Others were already in the form of a network, on which we could directly consider threshold contagions and shortest paths.

We analyzed the activation times in multiple realizations of a threshold contagion directly in terms of dimensionality, topology, and geometry, and we also did the same analysis after performing the *p*_dist_-operation, that is, after mapping data points to points in high-dimensional space based on these activation times. In doing so, we have added to what had been done with the original contagion map algorithm in Taylor et al. (2015) and Mahler (2021), which only examined point clouds in high-dimensional space. We studied these variants of the original contagion-map algorithm, and we did the analogous for Isomap, which, in its original form, only considered embedding dimension based on the shortest-path lengths directly. By comparing the variants of contagion maps and Isomap, we found not only that contagion maps perform better in many cases where Isomap breaks down due to noise-induced short circuit errors, but also that processing the distance estimates *via* the *p*_dist_-operation before analyzing them leads to clearer results. Indeed, we saw in some instances that this operation seems to accentuate our results (see e.g., the red data points in Figure 10A vs. Figure 10B, or Figure 11). Even more remarkably, in our method for determining topological features of a data set, the *p*_dist_ operation does not only emphasize results but seems, in some cases, to be a necessary methodological step, bringing out features that are not detectable without this pre-processing step (see Figures 12, 13). How exactly *p*_dist_ transforms a dissimilarity matrix and what effect this operation has on our various measures of geometry, topology, and dimensionality will be studied in future work.

## Data Availability Statement

The original contributions presented in the study are included in the article, further inquiries can be directed to the author.

## Author Contributions

The author confirms being the sole contributor of this work and has approved it for publication.

## Conflict of Interest

The author declares that the research was conducted in the absence of any commercial or financial relationships that could be construed as a potential conflict of interest.

## Publisher's Note

All claims expressed in this article are solely those of the authors and do not necessarily represent those of their affiliated organizations, or those of the publisher, the editors and the reviewers. Any product that may be evaluated in this article, or claim that may be made by its manufacturer, is not guaranteed or endorsed by the publisher.

## Footnotes

^1^Note that, as the “weights” in this graph are really distances, a small weight of an edge indicates a strong connection between its incident nodes.

^3^The length of a shortest path between nodes *i* and *j* is denoted by *d*_G_(*i, j*). Note that the function *d*_G_:*V* × *V* → [0, ∞) is a metric on the set of nodes or (equivalently) on the set of data points.

^4^ https://web.mit.edu/cocosci/isomap/datasets.html

^5^ http://appliedtopology.github.io/javaplex/
